# The Holocene temperature conundrum answered by mollusk records from East Asia

**DOI:** 10.1038/s41467-022-32506-7

**Published:** 2022-09-02

**Authors:** Yajie Dong, Naiqin Wu, Fengjiang Li, Dan Zhang, Yueting Zhang, Caiming Shen, Houyuan Lu

**Affiliations:** 1 Key Laboratory of Cenozoic Geology and Environment, Institute of Geology and Geophysics, Chinese Academy of Sciences, Beijing, 100029 China; 2 Innovation Academy for Earth Science, Chinese Academy of Sciences, Beijing, 100029 China; 3 Center for Excellence in Life and Paleoenvironment, Chinese Academy of Sciences, Beijing, 100044 China; 4 College of Earth and Planetary Sciences, University of Chinese Academy of Sciences, Beijing, 100049 China; 5 Yunnan Key Laboratory of Plateau Geographical Processes and Environmental Changes, Faculty of Geography, Yunnan Normal University, Kunming, 650500 China

**Keywords:** Atmospheric dynamics, Climate and Earth system modelling, Palaeoclimate, Palaeoecology

## Abstract

Seasonal biases (the warm-season contribution) of Holocene mean annual temperature (MAT) reconstructions from geological records were proposed as a possible cause of the mismatch with climate simulated temperature. Here we analyze terrestrial mollusk assemblages that best reflect seasonal signals and provide quantitative MAT and four-season temperature records for northern China during the past 20,000 years. The MAT estimated from the seasonal temperatures of a four-season-mean based on mollusks shows a peak during ~9000–4000 years ago, followed by a cooling trend. In general, the contribution of summer and winter temperature to MAT is significantly greater than that of spring and autumn temperatures. The relative contribution of each season varies over time and corresponds roughly with the seasonal insolation in each season. This independent evidence from mollusk records from the mid-latitudes of East Asia does not support the Holocene long-term warming trend observed in climate simulations and the seasonal bias explanation.

## Abstract (web summary)

Scientists have been puzzled by the disparity between climate simulations of the past 12,000 years and geological records. Dong et al. reconstructed past annual and seasonal temperatures from land snail records to examine the potential seasonal bias.

## Introduction

The long-term evolution of hemispheric and global temperatures during the Holocene is controversial^1–6^ because proxy and model data show diverging temperature trends between different reconstructions. Transient climate models revealed monotonic warming temperatures throughout the Holocene^1,3^, which seem to be supported by quantitative reconstructions of winter seasonal temperature at several regions at the high latitudes of continental Eurasia^4,7,8^. However, proxy reconstructions based on a stack of globally distributed temperature records (~80% marine records) indicated an early to middle Holocene Thermal Maximum (HTM; ~10 to 6 ka) in the Northern Hemisphere and subsequent cooling during the late Holocene^2^. This proxy-model discrepancy has been dubbed the ‘Holocene temperature conundrum’^3^, and the ultimate cause remains unclear.

Several studies in simulation research proposed that the MAT of proxy-based climate reconstructions may represent the temperature of certain months, or of a particular season^3,6,9^. Additionally, the existence of a Holocene thermal maximum was dominated by the temperature of the warm season^6,7^.

For paleorecords, only a few proxy-based-temperature reconstructions were used to quantify seasonal or monthly parameters, which limits our understanding of the contribution of monthly and seasonal temperatures to the MAT during the Holocene. Therefore, selecting a temperature-sensitive proxy to test the relationship between the proxy and seasonal temperature, determining whether or not the averaged seasonal or monthly temperatures show the same trend as the MAT computed in an independent way, and clarifying the contribution of each seasonal temperature to the MAT, are potentially one means of resolving the ‘Holocene temperature conundrum’.

**Fig. 1:**
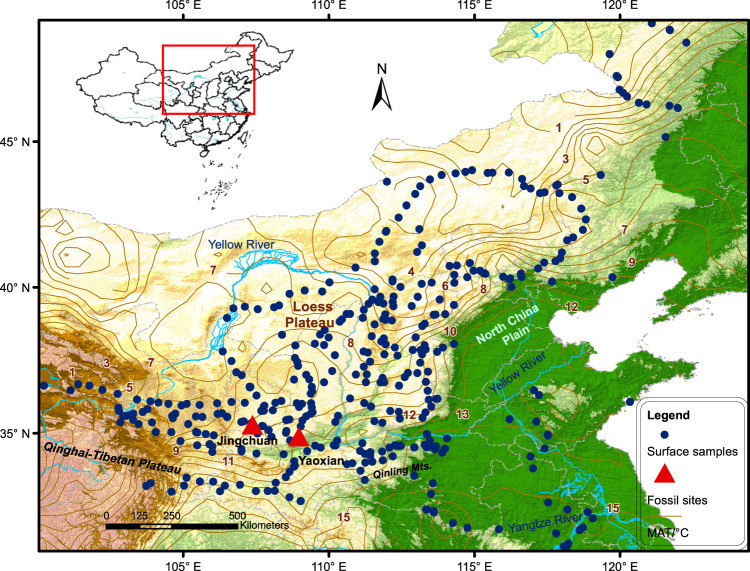
Map of the study area in northern China showing the locations of the 382 surveyed plots (blue dots) and the studied loess profiles (red triangles). The grey lines are isotherms (MAT/°C). Insert shows the location of the study region in China. The base map of this figure was generated using DIVA-GIS 7.5 (http://www.diva-gis.org/).

Terrestrial biological assemblages have great potential for reconstructing the average temperature in different seasons and the MAT. Terrestrial mollusks are sensitive to seasonal temperature changes^10^ and they are widely distributed and well preserved in Quaternary sediments^10–12^. Here, they were selected to clarify the relationship between species assemblages and seasonal temperature in northern China. First, 382 surface soil mollusk samples were systematically investigated covering a wide climatic gradient, ranging from ~ −23 to 5.9 °C and from ~10.9 to 27.9 °C for the coldest and warmest seasonal temperatures^13^, respectively (Fig. 1, Supplementary Table 1). A quantitative mathematical model of seasonal temperatures and MAT was then developed using a transfer function based on the modern mollusk-climate datasets. We applied the mollusk-climate calibration model to two fossil mollusk records spanning the last 20,000 years from the Chinese Loess Plateau (CLP), which represents the history of seasonal temperature changes in the East Asian monsoon region.

Our aims were: (1) to test the potential of mollusk assemblages for reflecting the average temperature in different seasons and the MAT; (2) to determine the long-term trend of the MAT estimated from the four seasons; and (3) to assess the contribution of different seasonal temperatures to the MAT over time, and to determine whether Holocene temperature reconstructions are biased towards a particular season.

## Results and discussion

### Modern and fossil mollusks as potential indicators of seasonal temperatures

**Fig. 2:**
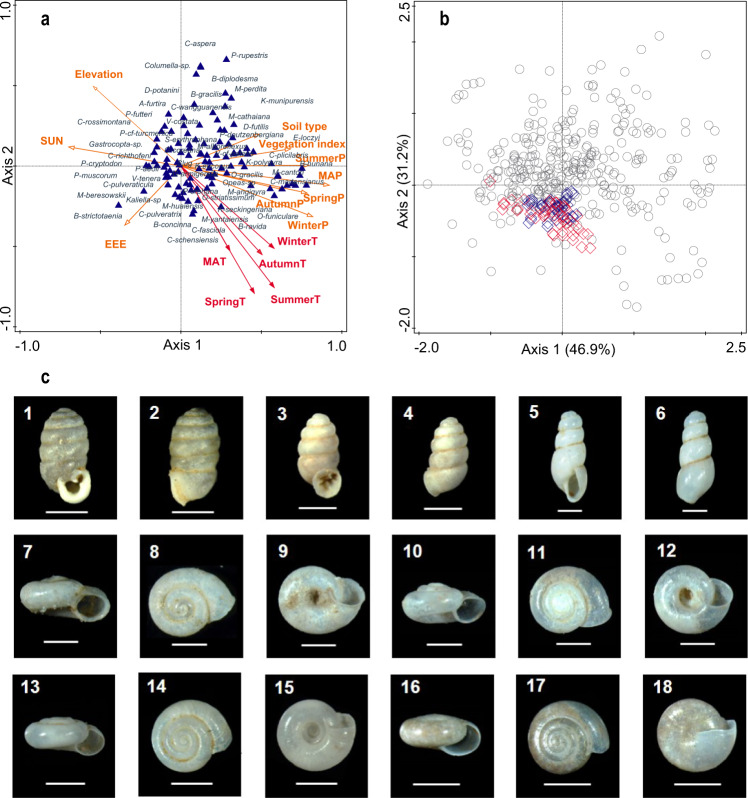
Statistical association of mollusk species with seasonal climate parameters. Results of canonical correspondence analysis (CCA) showing the effects of seasonal temperatures (red arrows) and other environmental factors (orange arrows) on mollusk species (blue triangles) (**a**), ordination diagram of NMDS of the modern mollusk data set (grey circles), and fossil samples at Yaoxian (blue diamonds) and Jingchuan (red diamonds) (**b**), based on Bray-Curtis dissimilarity distances, and photos of representative mollusk species in the Yaoxian and Jingchuan loess-palaeosol sequences (**c**). All scale bars are 1 mm. MAT mean annual temperature, MAP mean annual precipitation, EEE annual evaporation, SUN sunlight times. Vegetation cover index represented by the NDVI (normalized difference vegetation index). 1–2 lateral and dorsal views of *Pupilla aeoli* from L_1−1_ loess; 3–4 lateral and dorsal views of *Gastrocopta armigerella* from the S_0_ paleosol; 5–6 lateral and dorsal views of *Opeas striatissimum* from the S_0_ paleosol; 7–9 lateral, apical and umbilical views of *Vallonia tenera* from the L_1−1_ loess; 10–12 lateral, apical and umbilical views of *Vallonia* cf. *pulchella* from the L_1−1_ loess; 13–15 lateral, apical and umbilical views of *Punctum orphana* from the S_0_ paleosol; 16–18 lateral, apical and umbilical views of *Macrochlamys angigyra* from the S_0_ paleosol.

We first present 19 potential environmental variables affecting terrestrial mollusk assemblages in order to explore their relationship and to identify the main environmental factors reflected by a modern mollusk dataset of 382 topsoil samples (Fig. 1). Using principal coordinates analysis (PCoA) and canonical correspondence analysis (CCA) ordination methods with the Monte Carlo permutation procedure (see *Methods*), and after removing high multicollinearity, 15 environmental variables were included in the CCA analysis (Fig. 2). The CCA revealed that 26.6% of the total variance in the mollusk data was explained by these 15 variables, including, for example, spring temperature, summer temperature, autumn temperature, winter temperature, mean annual temperature (MAT), mean annual precipitation, soil type, and vegetation index (Fig. 2, Supplementary Table 2). The relevance of these variables for explaining changes in mollusk species composition is demonstrated by the highly significant correlations between site scores on the first two PCoA axes (using the entire variance of the species data) (Supplementary Fig. 1, Supplementary Table 3) and the CCA (using only the variance explained by the given factors) (Fig. 2, Supplementary Table 2). The PCoA results show that the site scores on the first and second axis are significantly associated with winter temperature, autumn temperature, MAT, spring temperature, and summer temperature (Supplementary Table 3). The CCA results also show that the effects of MAT and four seasonal temperatures on the modern snail assemblages are statistically significant at the *p* < 0.05 level, and that even the effects of seasonal temperature are more significant than that of MAT (Fig. 2, Supplementary Table 2). The arrow directions of the temperature gradients in Fig. 2 point to the southern region with high seasonal temperatures, where *Punctum orphana, Gastrocopta armigerella, Opeas striatissimum* are abundant^10,12,14^, implying a significant turnover in species occurrence and abundance in response to seasonal temperature changes in the study area.

Ecologically, spring and summer temperatures directly determine the occurrence and abundance of mollusk species, while winter-spring temperatures determine the advance and delay of snail hibernation^14^, i.e., the length of the snail growing season^15^, and thus they indirectly affect the emergence and abundance of individual snail species^16,17^. This is not unexpected since the study area is located in mid-latitudes, with a large temperature gradient during the winter months. Furthermore, other biological assemblages (e.g., vegetation) that are closely related to the mollusk food chain have been suggested to be significantly influenced by winter temperature^18^. In this way, mollusks that exhibit ~2–3 years’ growth or grow in a single year all incorporate biological imprints of the seasonal temperatures. This is confirmed by observations of different periods of activity in modern mollusks, ranging from late spring (e.g., *Pupilla aeoli, Bradybaena ravida*) to mid-summer (e.g., *P. orphana, Cathaica pulveratrix*), and even into early autumn^19–21^. Thus, snail assemblages and their abundance in topsoil can reveal seasonal and annual temperature changes in terms of both statistical and ecological criteria.

The two loess-palaeosol sections of Yaoxian (34°53′N, 108°58′E, 673 m a.s.l.) and Jingchuan (35°15′N, 107°43′E, 1244 m a.s.l.) are located in the southern and central Chinese Loess Plateau (CLP) (Fig. 1), which is characterized by four distinct seasonal temperature regimes. Seasonal temperature became a primary control on the occurrence and abundance of mollusks in the region^10–12^. A total of 230 fossil assemblage samples from the two sections, representing 28 species, reveal the succession of snail assemblages during the past 20,000 years^22^ (Supplementary Figs. 2–4). The dominant mollusk species present in fossil and modern samples are located on both sides of the first CCA axis. Non-metric multidimensional scaling (NMDS) ordination based on the Bray-Curtis dissimilarity distance was conducted to represent the locations of fossil samples within the data cloud of the modern mollusk samples (Fig. 2b). The results indicate that all fossil samples have good modern analogues. A goodness-of-fit analysis also showed that over 95% of the fossil samples have either good or fair analogues (Supplementary Fig. 5), indicating an overall good match between the fossil and modern snail samples.

The foregoing ecological and statistical analyses of a modern mollusk dataset consisting of topsoil samples, which accumulated over multiple years or decades, reveal significant quantitative relationships between mollusk assemblages and 50-year averages of seasonal and monthly temperature. Theoretically and logically, these quantitative relationships can be applied to fossil samples, which accumulated over ~200–300 years (the time interval represented by a sample), to reconstruct seasonal temperatures. This proven method has been widely applied to reconstruct the mean temperature in January and July during the geological past in Europe using mollusk records^16,17,23,24^, as well as in Europe and East Asia using pollen, midge, and phytolith records^25–30^. It is evident, therefore, that both modern and fossil mollusks can serve as potential indicators of seasonal temperatures.

### MAT and seasonal temperature reconstructions since the LGM

The modern snail samples were then used as an independent training set to develop transfer functions for MAT and seasonal temperature reconstructions. Locally-weighted weighted-averaging partial least squares (LW-WAPLS) regression, weighted-averaging partial least squares (WAPLS) regression, and locally-weighted weighted averaging (LW-WA) regression were chosen as a unimodal model. Comparison of these mollusk-temperature calibration models indicated that the LW-WAPLS calibration model performance was the most statistically significant, as indicated by the high *R*^2^ value, low RMSEP, and average and maximum biases (Supplementary Fig. 6, Supplementary Table 4). Thus, the LW-WAPLS model for seasonal temperatures was selected for the reconstructions. A reconstruction significance test^31^ showed that all seasonal temperatures and MAT for all of the records explained more than 95% of the variance of all the random reconstructions (Supplementary Fig. 7), suggesting that the selected LW-WAPLS model is statistically significant for reconstructing MAT and season-by-season temperature.

**Fig. 3:**
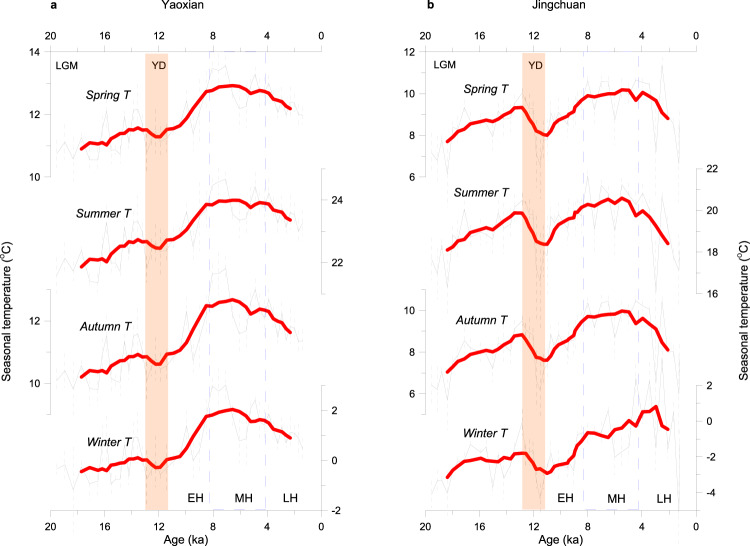
Regional seasonal temperature reconstructions based on mollusks. 20,000-year reconstruction of seasonal temperature from the loess sequences at Yaoxian (**a**) and Jingchuan (**b**) on the Chinese Loess Plateau. The raw data are overlain with the results of the application of a smoothing function (LOWESS) and the uncertainty in the reconstruction based on mollusks is shown by the light-grey error envelope (±1 SD). Temporal intervals of the last glacial maximum (LGM), Younger Dryas (YD) (red shaded bar), early Holocene (EH), mid-Holocene (MH) (blue box), and late Holocene (LH) are indicated.

**Fig. 4:**
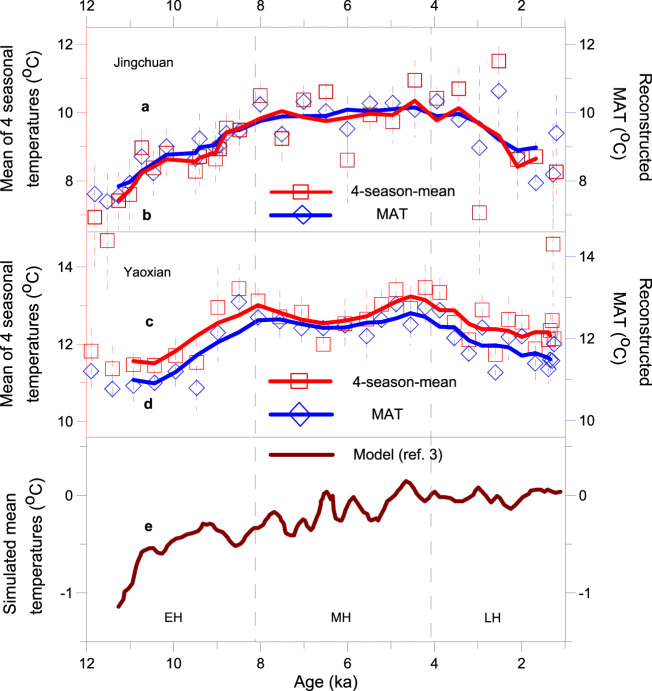
Evolution of Holocene mean annual temperature (MAT) in the Chinese Loess Plateau. Holocene mean-four-season temperature (red squares and lines) (**a**, **c**) from the loess sequences, and comparison with a direct reconstruction of MAT (blue diamonds and lines) (**b**, **d**) and simulated mean temperatures^3^ (brown line) (**e**). The raw data are overlain with the results of the application of a smoothing function (LOWESS) and the uncertainty in the reconstruction is shown by the dotted error envelope (±1 SD). The early Holocene (EH), mid-Holocene (MH), and late Holocene (LH) intervals are indicated.

Our reconstructions from fossil snail records (Supplementary Figs. 2–4) of two loess profiles in the Chinese Loess Plateau (CLP) show a trend of increasing spring, summer, autumn, and winter temperatures during the last deglaciation, a temperature peak during the early to mid-Holocene, and decreasing temperatures during the late Holocene (Fig. 3). This pattern is also revealed by the MAT estimated from season-by-season temperature, as well as by the MAT reconstructed independently by the LW-WAPLS transfer function (Fig. 4). There are some slight differences between the two sites in terms of the amplitude and time range of the Holocene thermal maximum, which may be related to the further northward location of Jingchuan, which delays the timing of the influence of the East Asian summer monsoon in the region^22^, as well as regional topography and other microenvironmental factors. Nevertheless, the overall pattern of the Holocene temperature optimum occurring at ~9–4 ka at the two sites is significant.

A similar pattern was revealed by a phytolith-based seasonal temperature reconstruction^30,32^, a physically-based climate proxy from the loess deposits of the Chinese Loess Plateau^33^, pollen-based temperature reconstructions from paleolimnological records from northeast China^34,35^ and from the coastal lowlands of eastern China^36^, and pollen records from Korea and Japan^37^, as well as marine records from the East China Sea^38,39^ and the western equatorial Pacific^40^, suggesting an early to middle Holocene thermal maximum in East Asia. These independent lines of evidence indicate a consistent early to middle Holocene thermal maximum in East Asia, suggesting that seasonal bias in the proxies may not be the primary cause of the Holocene proxy-model mismatch, at least in East Asia.

In addition, the spatial heterogeneity of temperature changes and the sensitivity of the proxies should be considered in explaining data-model discrepancies, because this factor may cause the timing and magnitude of Holocene warming to vary across regions at different latitudes. For example, the brGDGTs-based Holocene MAT reconstruction from lakes in the Tibetan Plateau revealed different temperature variations during the middle Holocene^41^. Even so, given the similarity of these cooling trends in the late Holocene, it appears unlikely that biological and/or physical proxies from the study area and from other regions of East Asia can universally be considered to be seasonally biased.

### Different seasonal contributions to MAT

**Fig. 5:**
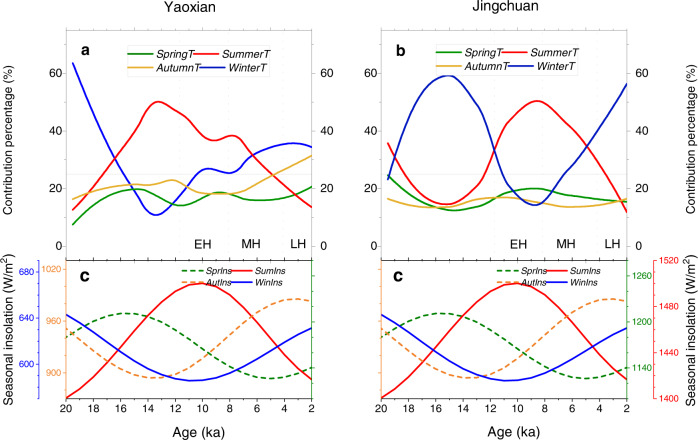
The contribution of different seasonal temperatures to the MAT. Variation of the relative contribution of mean temperature in different seasons to the MAT over time at Yaoxian (**a**) and Jingchuan (**b**) during the Holocene, and their relationship with seasonal insolation^44^ (**c**). The early Holocene (EH), mid-Holocene (MH), and late Holocene (LH) intervals are indicated.

The key aspect of the Holocene temperature conundrum is clarifying the contribution of different seasonal temperatures to the MAT. We estimated changes in the contribution percentage of different seasonal temperatures to the MAT during the Holocene using the Variance Contribution method (see *Methods*)^42,43^. Temporally, the relative contribution of seasonal temperatures to the MAT varied during the Holocene. The contribution of summer warming to the MAT was greater than that of other seasons in the early Holocene (Fig. 5), which is in line with the higher summer insolation. However, the relative influence of winter temperatures increased substantially during the middle and late Holocene, which coincided with a significant increase in winter insolation (Fig. 5).

According to the overall frequency distribution of the contribution percentage of four seasons during the past 20,000 years, the contribution of winter and summer temperatures to the variation of MAT is significantly greater than that of spring and autumn temperatures (Supplementary Fig. 8), since the former two have a large deviation from the MAT (Supplementary Fig. 9). Warm season temperature is proposed to dominate the Holocene temperature pattern based on proxy reconstructions^6^, and our records indicate that the contribution of winter temperature was not negligible, although it is often ignored or underrepresented in proxy reconstructions^8^. Indeed, winter temperature (or reconstructed monthly temperatures in winter months) shows a large range of variation and increased by ~5–6 °C from the last deglaciation to the early to mid-Holocene in mid-latitude East Asia (Fig. 5, Supplementary Figs. 10–12). The significant contribution of winter and summer temperatures to the MAT is also shown in the instrumental records of the two sites during the past 50 years (Supplementary Figs. 13, 14).

Note that implicit in transient climate models is that winter temperatures follow winter insolation, and summer temperatures follow summer insolation^3,6^. Our records show that the temporal evolution of winter and summer temperature is not completely anti-phased as expected, indicating that the seasonal temperature does not simply respond to winter and summer insolation as predicted by climate models^1,3^. The similarity of annual and seasonal temperature patterns to summer insolation^44^ in the Northern Hemisphere indicates that summer insolation in the Northern Hemisphere may be the initial driver of climate change in mid-latitude East Asia, via ice sheet effects, combined with the feedback of more complex factors such as monsoon circulation (via precipitation-induced cloud cover change), atmospheric dust, and vegetation^45–47^. Against this background, our records indicate that the relative contribution of seasonal temperature over time was further modified by the seasonal insolation.

This in turn suggests that uncertainty in transient climate models may also give rise to data-model inconsistencies. Chains of climate feedbacks, such as summer monsoon–vegetation–dust aerosol–clouds, linked by summer insolation, are typically not well assessed and quantified in current transient climate models^46–49^. Simulations with prescribed changes in vegetation and dust show that biophysical feedbacks associated with vegetation changes reinforce Northern Hemisphere warming and monsoon precipitation during the mid-Holocene, reducing model-data discrepancies in the regional climate^46,47,50–52^. Additionally, changes in sea level, sea ice, and the land-sea geography of boundary conditions need to be specified for the paleoclimate experiments to quantify the uncertainties^53,54^.

Overall, our record provides a typical geological case for addressing the controversy between reconstruction and simulation from the perspective of the four seasons. Our results imply that the transient modeled Holocene monotonic increase in global average temperature was not universal across terrestrial records, at least in northern China. This also suggests that Holocene temperature reconstructions are not universally biased towards a particular season. This highlights the need for caution in directly comparing various regional temperature change modes from reconstructions with transient simulations for understanding past global climate change^1,3,4,6^. Our new records, however, reaffirm the importance of distinguishing and reconstructing seasonality in paleorecords, in order to better understand the long-term trends and dynamics of climate mean status and seasonal evolution.

## Methods

### Modern snail samples and fossil snail records from loess profiles

The surface-soil snail dataset used here was initially compiled by Li et al.^55^ and Dong et al.^56,57^, comprising 382 assemblages collected from surface soils across a ~1000 km climatic gradient in northern China (Fig. 1). Most of the samples were collected from natural habitats far from areas of human activity. Individual mollusks were selected using a combination of a visual search and extraction from a 2–3 cm-thick sample of litter and soil at each site^58^.

The two loess-palaeosol sections of Yaoxian (34°53′N, 108°58′E, 673 m a.s.l.) and Jingchuan (35°15′N, 107°43′E, 1244 m a.s.l.) are located in the southern and central CLP (Fig. 1). They are geographically located in ‘Yuan’ areas (flat-topped loess highlands, covered with thick loess deposits), one of the major topographic units in the CLP^10^. Sampling was undertaken on (i) the upper part of loess unit L_1_ (L_1−1_) which was deposited during and after marine isotope stage 2 (MIS2); and (ii) the lower part of the Holocene palaeosol unit (S_0_). The chronology of the two loess sequences is based on 24 optically stimulated luminescence (OSL) ages (Jingchuan, *n* = 9; Yaoxian, *n* = 15)^59^ (Supplementary Fig. 3). Each profile was sampled at 3-cm intervals, equivalent to an average temporal resolution of ~200–300 years per sample. A total of 230 samples were taken from L_1_ and S_0_, and all samples were washed and sieved in the field using a 0.5-mm mesh sieve to remove fine soil (Supplementary Fig. 2). The mollusk shells were then picked and identified to species level under a binocular microscope (Supplementary Fig. 2). Mollusk taxa data from the two loess sites were previously published by Dong et al.^22^ (Supplementary Fig. 4). All of the specimens were stored in the Key Laboratory of Cenozoic Geology and Environment, Institute of Geology and Geophysics, Chinese Academy of Sciences, China.

### Statistical analysis

To explore the effect of the temperatures of each season on the compositional variability of the snail data, principal coordinates analysis (PCoA) and canonical correspondence analysis (CCA) were used^60^. First, PCoA based on Bray-Curtis dissimilarity distances was conducted, producing biologically relevant dissimilarities, to explore the entire compositional variation among the samples. Target environmental variables were correlated to the ordination space to determine whether they were associated with the main compositional turnover, i.e. whether seasonal temperature was the main driver of the observed variation in the modern mollusk data. Direct ordination was also used to constrain the species variation to only that part that could be explained by the predictors, in order to determine their exclusive contributions as well as the amount of shared variation. Prior to this, detrended correspondence analysis (DCA) was used to calculate the gradient length of compositional turnover in standard deviation (SD) units. Our snail compositional data have a gradient >5 SD long, so a unimodal model performed by CCA analysis was appropriate. CCA analysis was then undertaken, and the variables were selected to be used after removing high multicollinearity until the Variance Inflation Factors (VIFs) were low. We used a combined model comprising a forward selection model to determine the uncorrelated variation, and a single model to test each variable separately. A combination of the two approaches, including only variables with an exclusive contribution to that subset to calculate the independent effect of each of the selected variables, enabled us to assess the marginal effects of all predictors and therefore how much variance was shared among them. The statistical significance of each variable was evaluated by Monte Carlo permutation tests with 999 permutations. All ordination analyses were run with square-root-transformed snail percentage data using the CANOCO program^61^.

### Transfer function reconstruction

Non-metric multidimensional scaling (NMDS) ordination was used to reveal the similarities between fossil and modern mollusk assemblages. The goodness of the matching between the calibration set and the fossil mollusk assemblages was also evaluated using the minimum dissimilarity between a fossil sample and the calibration set samples. Distances smaller than the 5th percentile of all distances between the training-set samples are considered to indicate good “analogues”, while distances larger than the 10th percentile are considered to indicate “no-analogue” assemblages. After the NMDS ordination and goodness-of-fit assessment, the good-quality calibration set selected by our framework provided a foundation for subsequent reconstruction.

Calibration models for season temperatures based on the fossil snail assemblages from two loess profiles in northern China were then developed using weighted-averaging partial least squares (WA-PLS) regression, locally weighted weighted-averaging (LW-WA) regression^62^, and locally-weighted weighted-averaging partial least squares (LW-WAPLS) regression^63^. The predictive performance of all calibration models was assessed by bootstrapping validation (validation of the model trained by the training-set of known climate parameters with the test set of the other examples), and performance statistics, including the root-mean-square error of prediction (RMSEP), the coefficient of determination (*R*^2^) between observed and predicted values, and the maximum bias in residuals were calculated for each calibration model.

Significance tests were used to assess the overall performance of the reconstructions for the entire fossil sequence, in addition to sample-specific error evaluation^31^. A number of random reconstructions (999 in this case) were derived from the training set. The proportion of the variance explained by these random reconstructions was estimated using redundancy analysis (RDA), a linear-based constrained ordination technique^64^. All calibration models were developed using C2^65^ and R software (version 4.1.3). Packages “Analogue” and “rioja” were used for WA, WA-PLS, Modern Analogue Technique, LW-WAPLS models^66,67^, and the package “PalaeoSig” was adopted for significant testing^68^.

### Seasonal contribution

The percentage distribution of seasonal contributions and their fluctuations over time were performed to separately evaluate the explanatory power of each seasonal variable for the reconstructed Holocene mean annual temperature. To avoid artifactual variations in season contribution caused by irregular temporal sampling resolution in the original mollusk sequences at different sites and to facilitate the comparison of different sites, the original seasonal temperature data since the LGM were first interpolated at equal time intervals of 100 years^4^. The temperature variance of each season and the sum of the four seasonal variances were then calculated at each 1000 yr interval (including 500 years before and after, approximately 10 sample sizes). The variance contribution of seasonal temperature [$Vi=(\frac{1}{n-1})\mathop{\sum }\nolimits_{1}^{n}{({Ti}-{Tm})}^{2}$] is used to evaluate the magnitude of temperature change (e.g. the magnitude of temperature increase or decrease) and the overall variation of the temperature in a specific time interval (1000 yr) relative to the mean temperature value of the season^42,43,69,70^, where *Vi* is the variance of temperature change in each season within this interval, *Ti* is the temperature of the ith sample in this season within this interval, and *Tm* is the mean temperature of a season within this interval. Since the MAT is the arithmetic average of all the seasonal temperatures^71^, a large seasonal variance denotes a significant increase or decrease in temperature change, which means that MAT fluctuations caused by this season will also be large^43,72^. The percentage contribution of a certain season in this interval is obtained by dividing the variance of the seasonal temperature by the sum of the four seasonal variances. We calculated the percentage contribution of each season for every 100 sliding years, and then (1) counted the frequency distributions of percentage contribution to clarify the overall contribution distribution of each season in the whole time series, and (2) smoothed the time series to analyze the trend of the relative influence of seasonal temperatures on MAT over time^73^.

### Reporting summary

Further information on research design is available in the Nature Research Reporting Summary linked to this article.

## Supplementary information

Supplementary Information

Reporting Summary

## Data Availability

The data to support all the analysis in this study have been deposited in the Zenodo repository (10.5281/zenodo.6426911)^74^.

## Code availability

The code to reproduce all the results in this study have been deposited in the Zenodo repository (10.5281/zenodo.6426798)^73^.
