# Supplementary material for: The Holocene temperature conundrum answered by mollusk records from East Asia
Source: Nat Commun. 2022 Sep 2;13:5153. doi: 10.1038/s41467-022-32506-7 (PMC9440108; doi:10.1038/s41467-022-32506-7)
Supplement: Supplementary file 1 — Supplementary Information [file 41467_2022_32506_MOESM1_ESM.pdf]

Supplementary information

The Holocene temperature conundrum answered by mollusk records from East Asia

Yajie Dong [1, 2, 3, 6] *, Naiqin Wu [1, 6]*, Fengjiang Li [1, 2, 3], Dan Zhang [1, 4], Yueting Zhang [1, 4], Caiming Shen [5] & Houyuan Lu [1, 4, 6]

This PDF file includes:

Supplementary Figures 1-14

Supplementary Tables 1-4

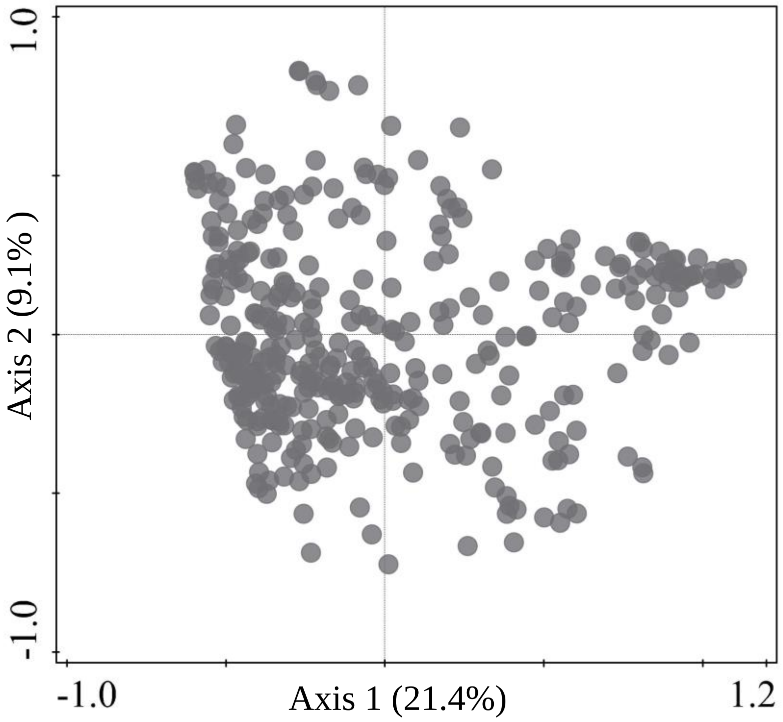

Supplementary Fig. 1. Ordination diagram of the results of principal coordinates analysis (PCoA) of the modern mollusk data set (grey circles), based on Bray-Curtis dissimilarity distances.

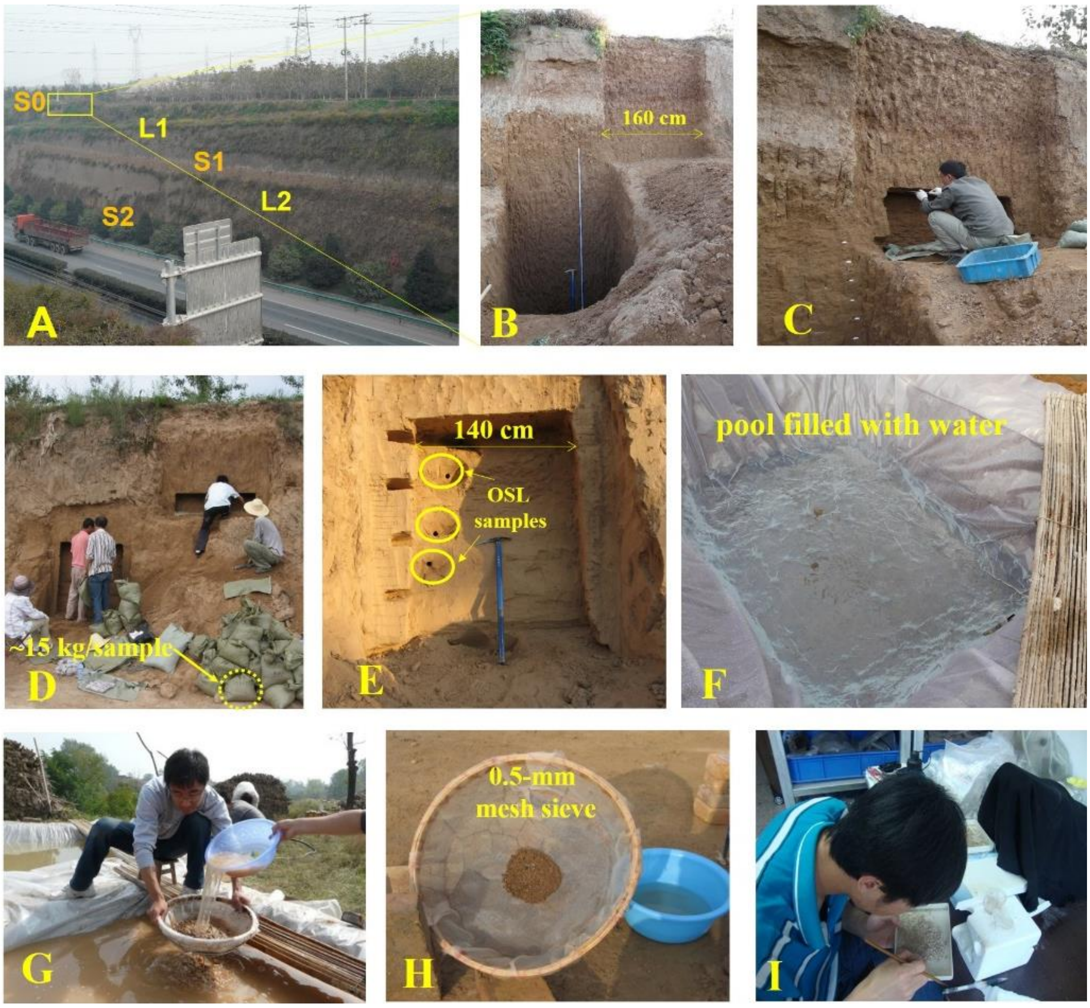

Supplementary Fig. 2. Photographs showing field sampling and the washing and sieving of mollusk fossil assemblages at each loess-palaeosol sequence.

Sampling of mollusk fossil assemblages from the same sampling column (3 cm × 140 cm × 40 cm) in the Yaoxian section (A–C) and Jingchuan section (D–E). Each sample weighed ~15 kg. The OSL samples were taken by hammering 20-cm-long stainless-steel tubes (5 cm diameter) into the freshly cleaned surface. All samples were washed and sieved in the field using a 0.5-mm mesh sieve (F-H). The mollusk shells were then picked and identified under a binocular microscope in the laboratory (I).

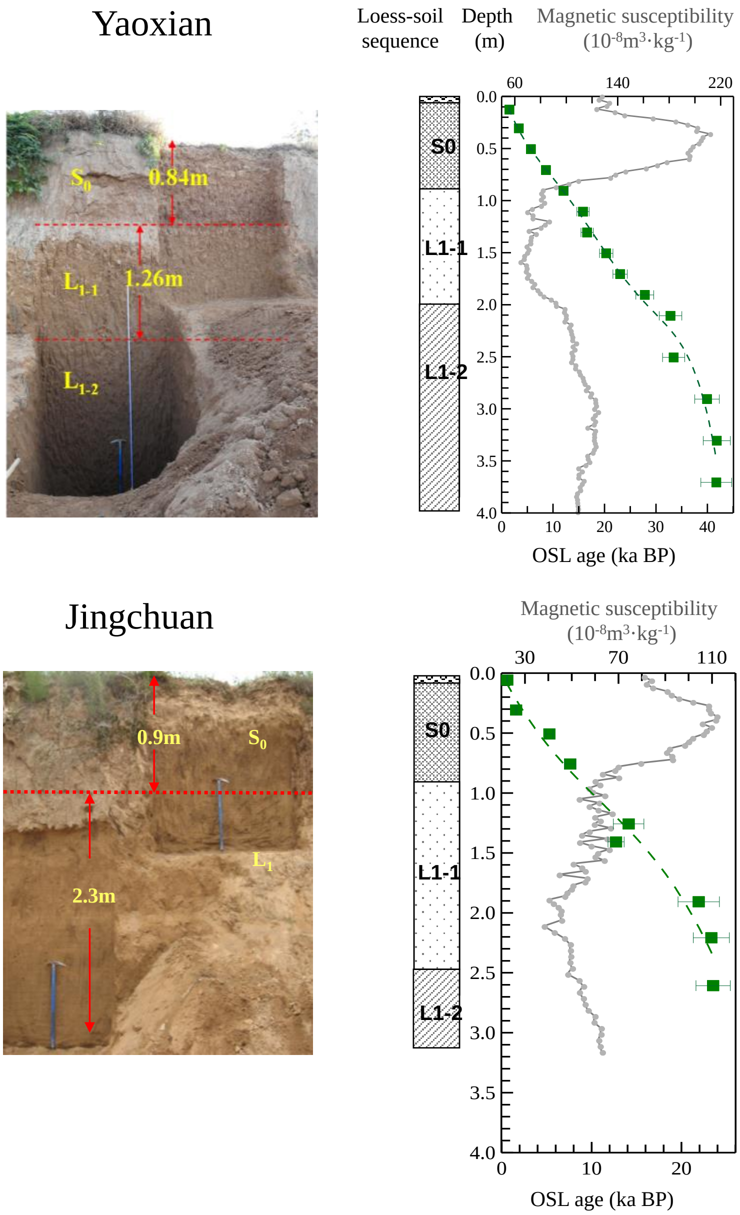

Supplementary Fig. 3. Field photographs, pedostratigraphy, magnetic susceptibility, and OSL ages versus depth for the loess sections at Yaoxian and Jingchuan since the last glacial maximum[59]. Dotted lines and solid arrows indicate the L1/S0 boundary and corresponding stratigraphic depths.

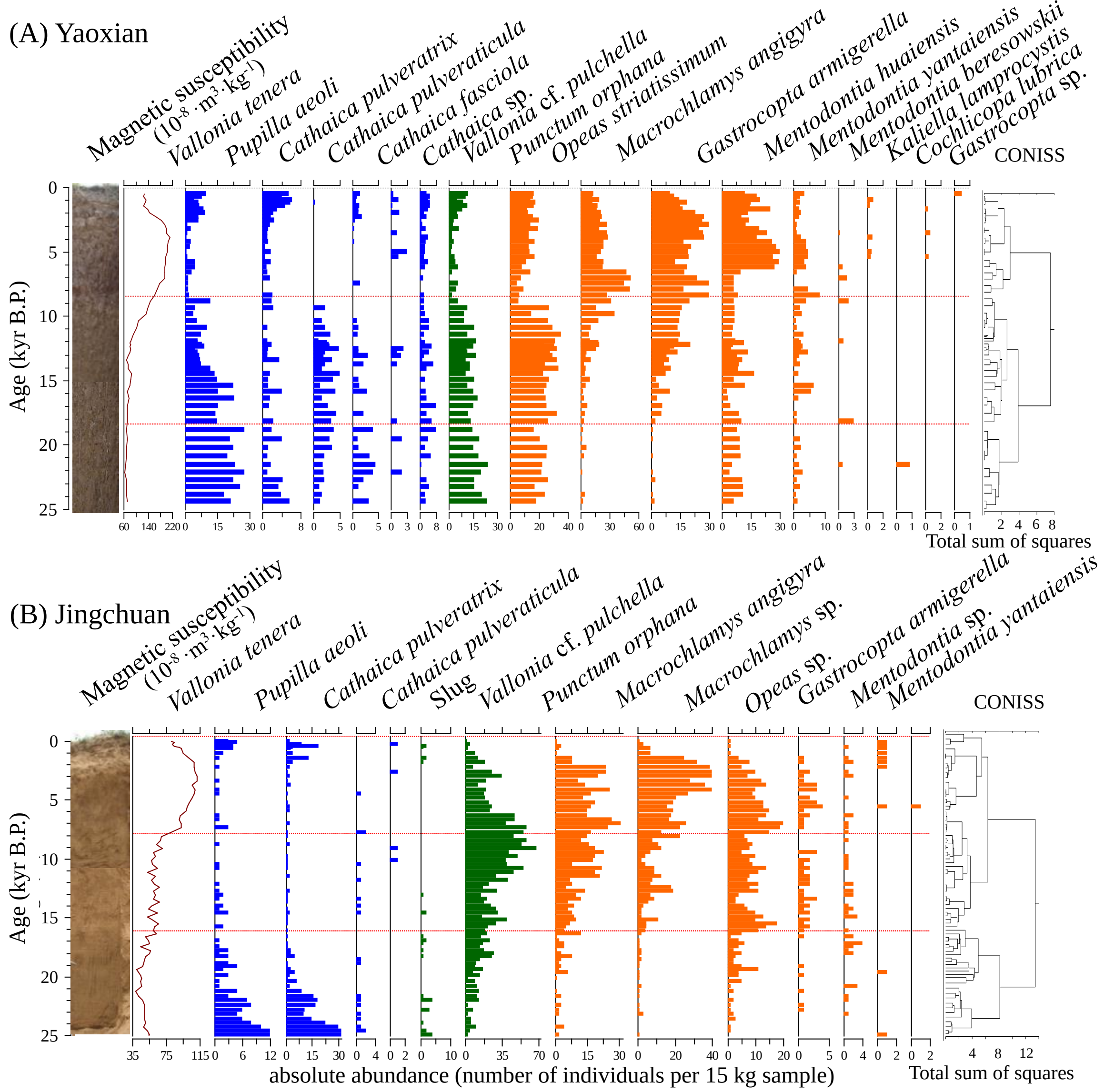

Supplementary Fig. 4. Variations of the absolute abundance of mollusk species (number of individuals per 15 kg of sediment) during the past 25 kyr at the Yaoxian (A) and Jingchuan (B) sections compared with the magnetic susceptibility record[22,59]. The three defined mollusk zones are based on the results of stratigraphically-constrained cluster analysis, which are shown on the right of the diagram.

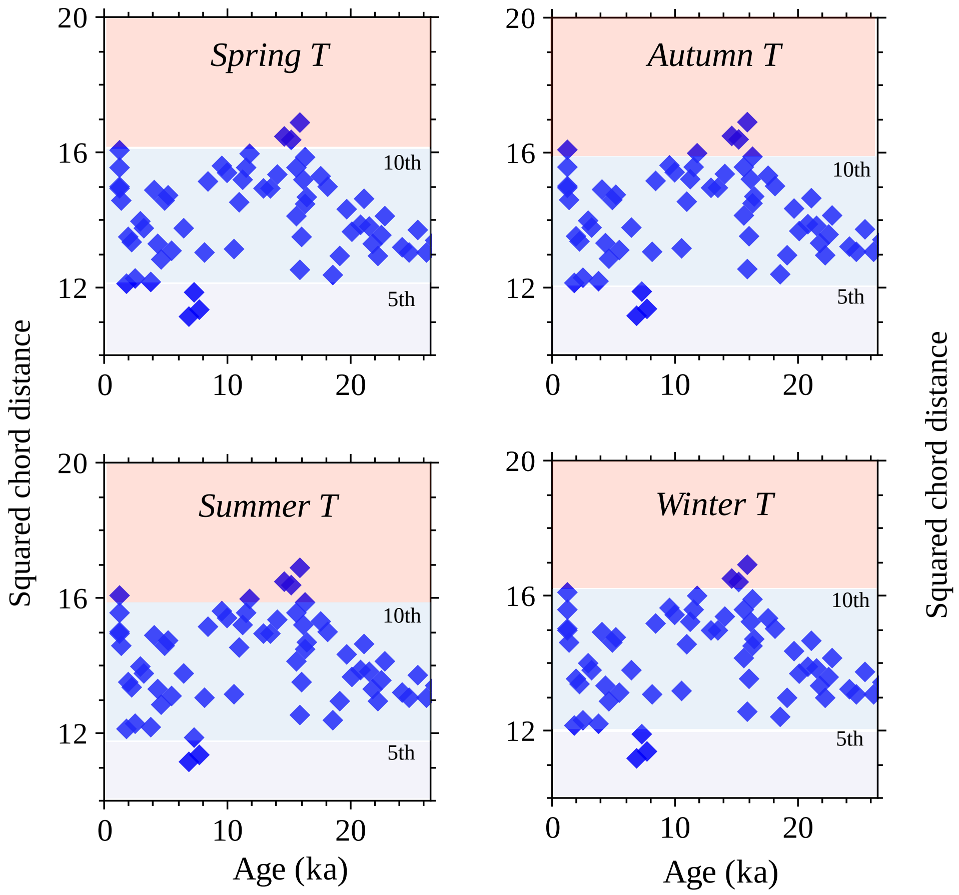

Supplementary Fig. 5. **Analogue quality for the reconstruction of the temperature of each season based on a goodness-of-fit analysis.** The gray and blue shadings show the 5th and 10th percentiles of the pair-wise distribution of squared-chord distances between the fossil samples and the best analogues from the modern training set, respectively. Distances smaller than the 5th percentile of all distances between the training-set samples are considered to be good "analogues", while distances larger than the 10th percentile are considered to be "no-analogue" assemblages.

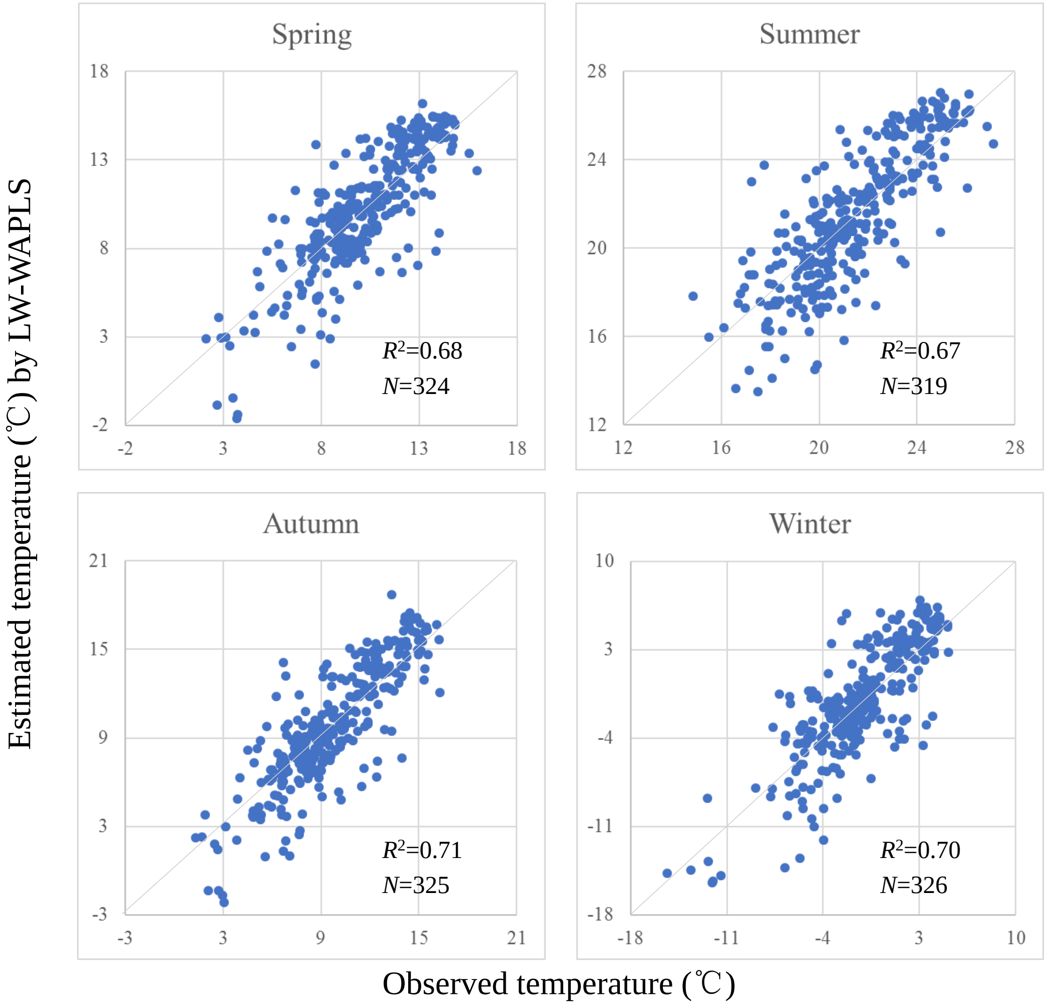

Supplementary Fig. 6. Scatter plots of observed versus LW-WAPLS predicted values for the temperatures of the four seasons in northern China. Observed values are on the x-axis and estimated values for the seasonal temperatures from the LW-WAPLS are on the y-axis. Each R^2 and N is shown in the lower right corner.

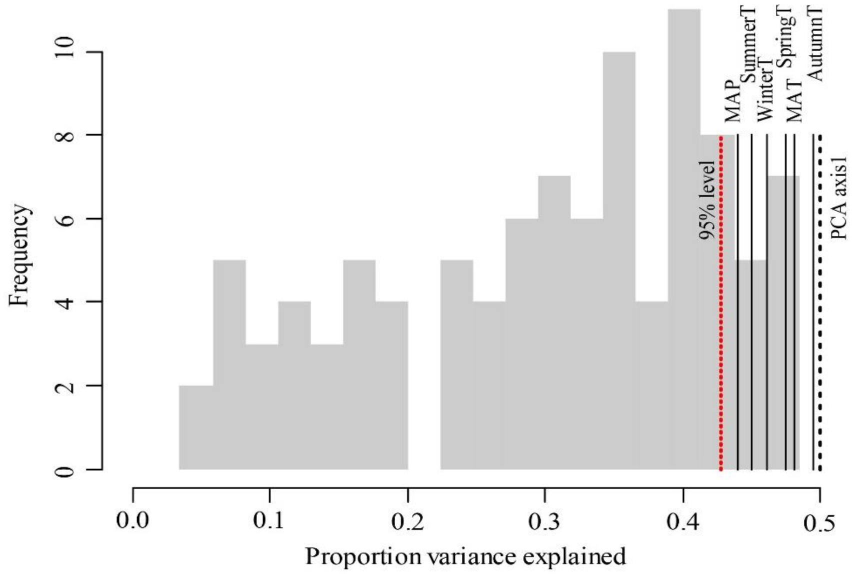

Supplementary Fig. 7. Significance test results for the mollusk-based climate reconstructions for the two loess sections. The red line represents the test line of the 95% significance level. The histogram in grey indicates the proportion of the variance. The black dotted line indicates the proportion of the variance explained by the first axis of a principal component analysis (PCA) of the fossil data.

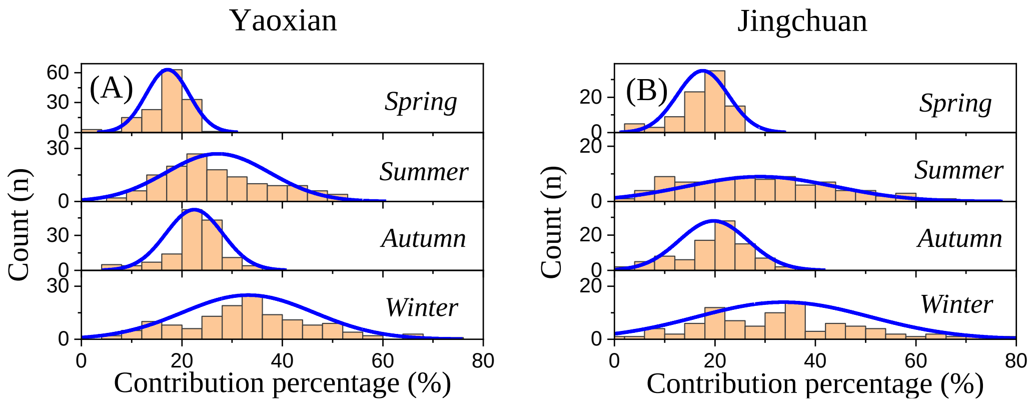

Supplementary Fig. 8. Frequency distributions of explanatory percentages showing the contribution of seasonal temperatures to the reconstructed MAT variation from the Yaoxian (A) and Jianchuan (B) loess profiles. The numbers on the horizontal axis are the percentage contribution of each season. The blue line represents the normal distribution curve.

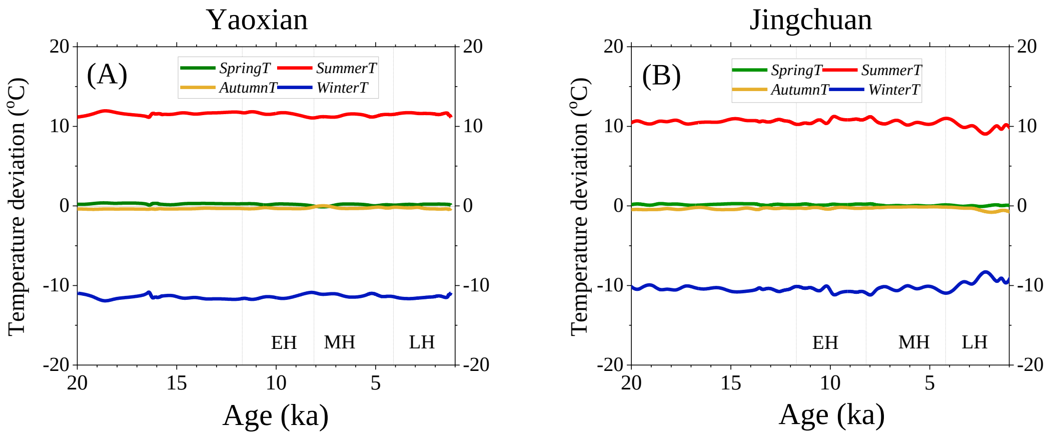

Supplementary Fig. 9. Variation of seasonal temperature deviation from the MAT over time at Yaoxian (A) and Jingchuan (B) during the past 20,000 years. The early Holocene (EH), mid-Holocene (MH), and late Holocene (LH) intervals are indicated.

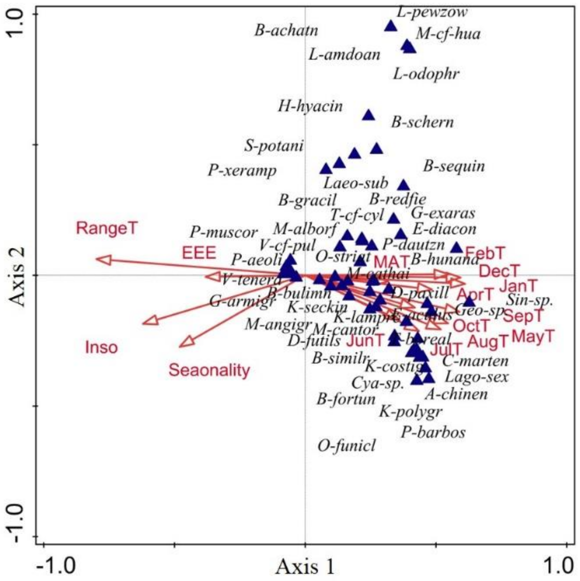

Supplementary Fig. 10. Results of canonical correspondence analysis (CCA) showing the effect of monthly temperatures (red arrows) and other environmental factors on mollusk species (blue triangles).

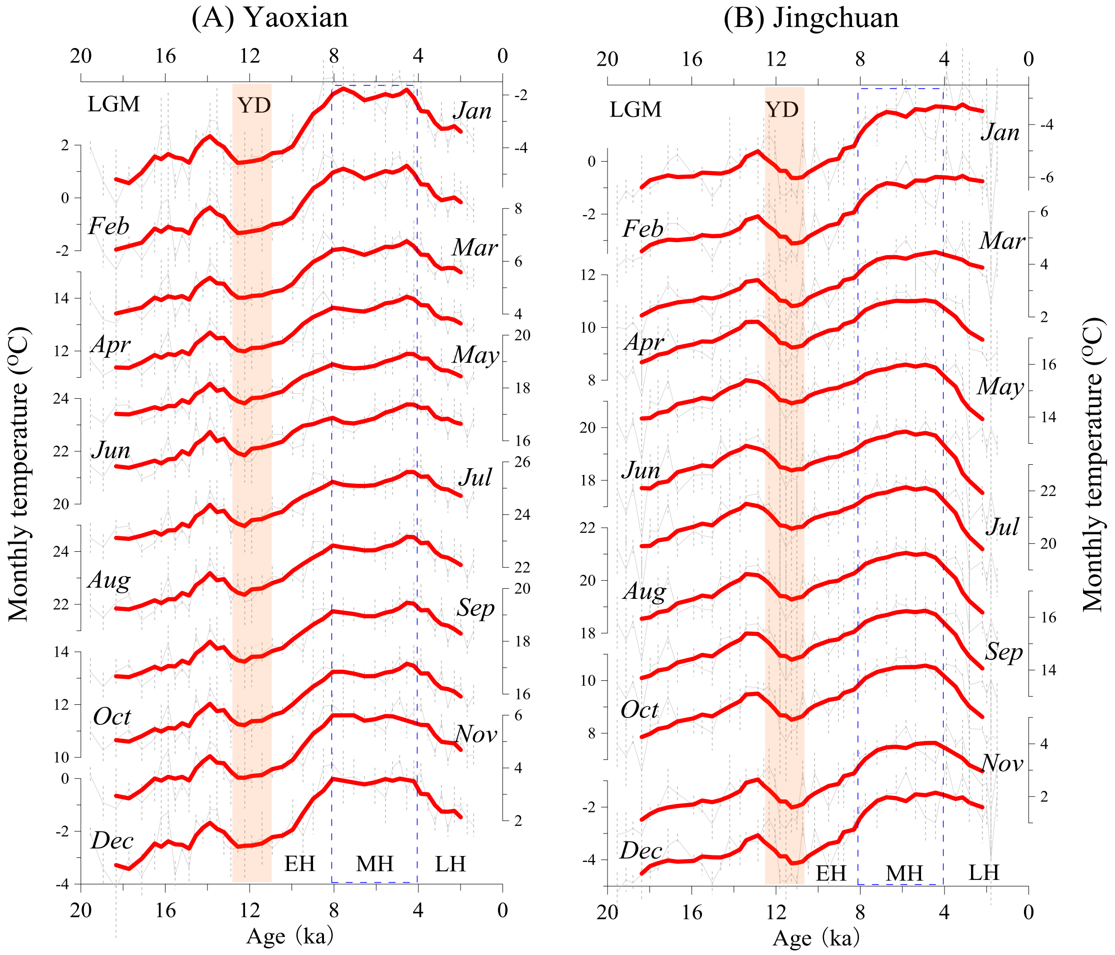

Supplementary Fig. 11. 20,000-year reconstruction of monthly temperature from the loess sequences at Yaoxian (A) and Jingchuan (B) in the Chinese Loess Plateau. The raw data are overlain with the results of the application of a smoothing function (LOWESS) and the uncertainty of the reconstruction is shown by the light grey error envelope (±1SD). Temporal intervals of the last glacial maximum (LGM), Younger Dryas (YD), early Holocene (EH), mid-Holocene (MH), and late Holocene (LH) are indicated.

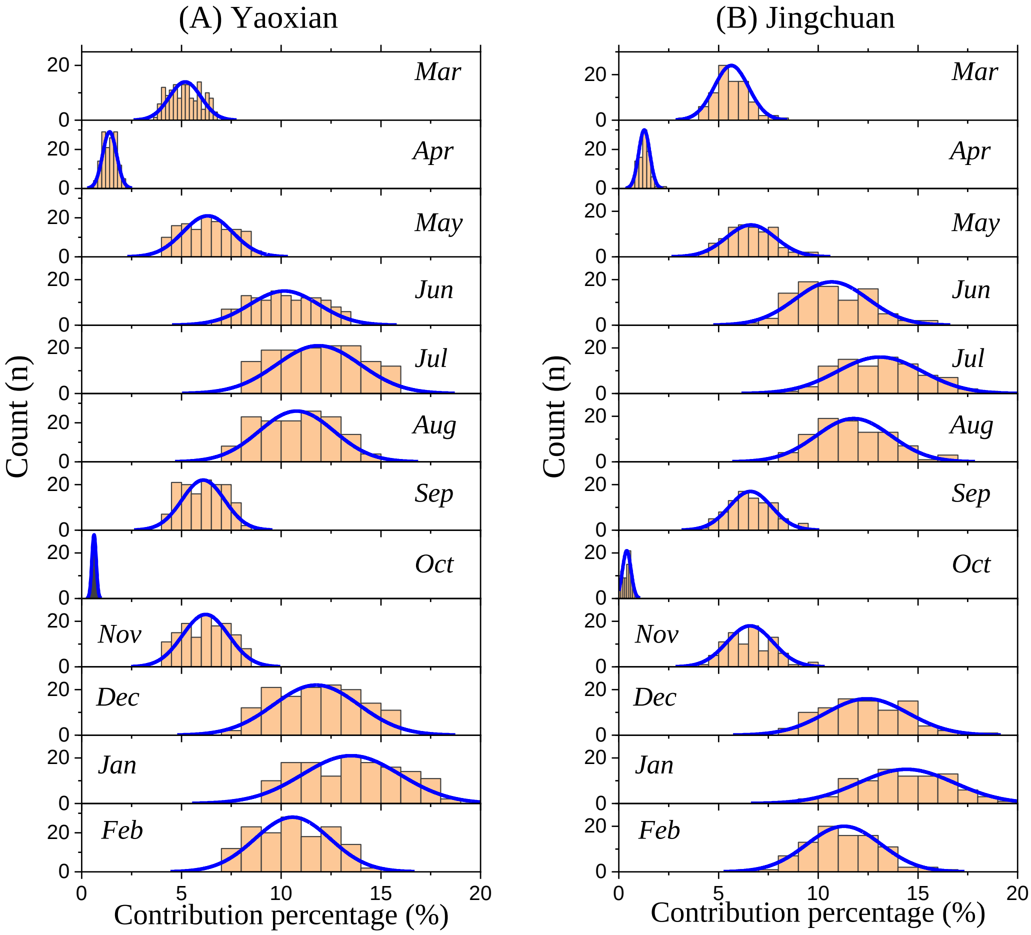

Supplementary Fig. 12. Frequency distributions of explanatory percentages showing the contribution of monthly temperature variables to the reconstructed MAT variations in the Yaoxian (A) and Jianchuan (B) loess profiles. The numbers on the horizontal axis are the percentage contribution of the temperature of each month. The blue line represents the normal distribution curve.

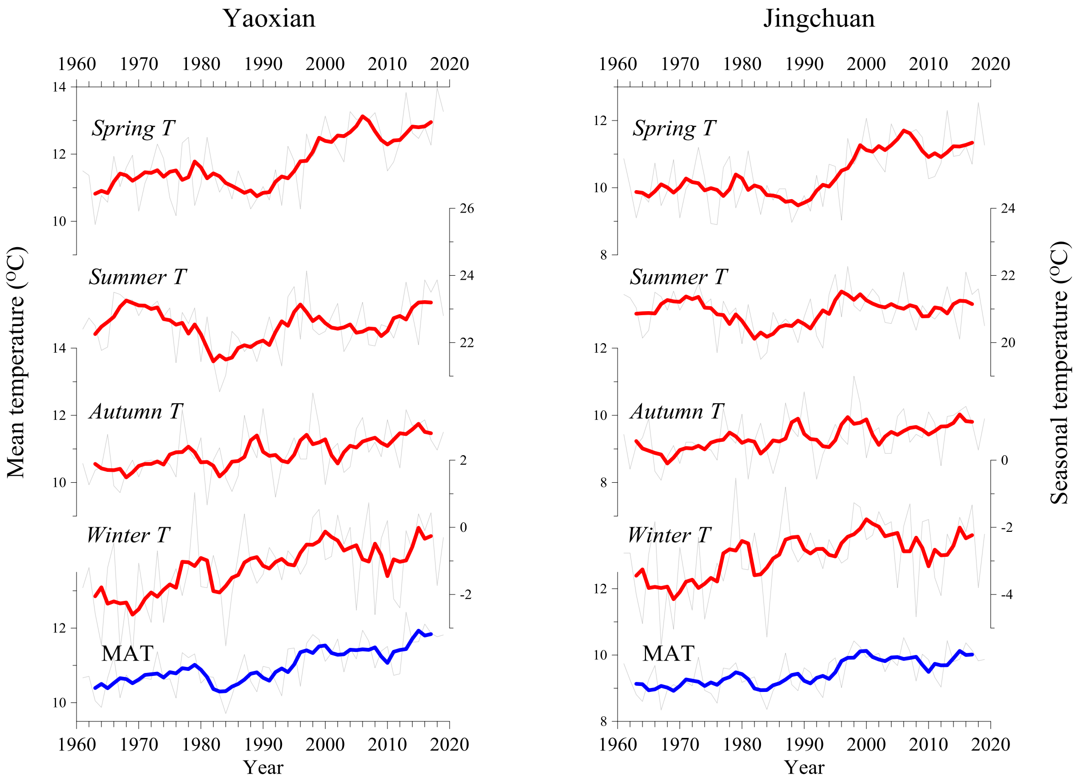

Supplementary Fig. 13. Seasonal temperature (red line) and mean annual temperature (blue line) at Yaoxian (A) and Jingchuan (B) site since the 1960. The raw data are overlain with the results of the application of a smoothing function (LOWESS).

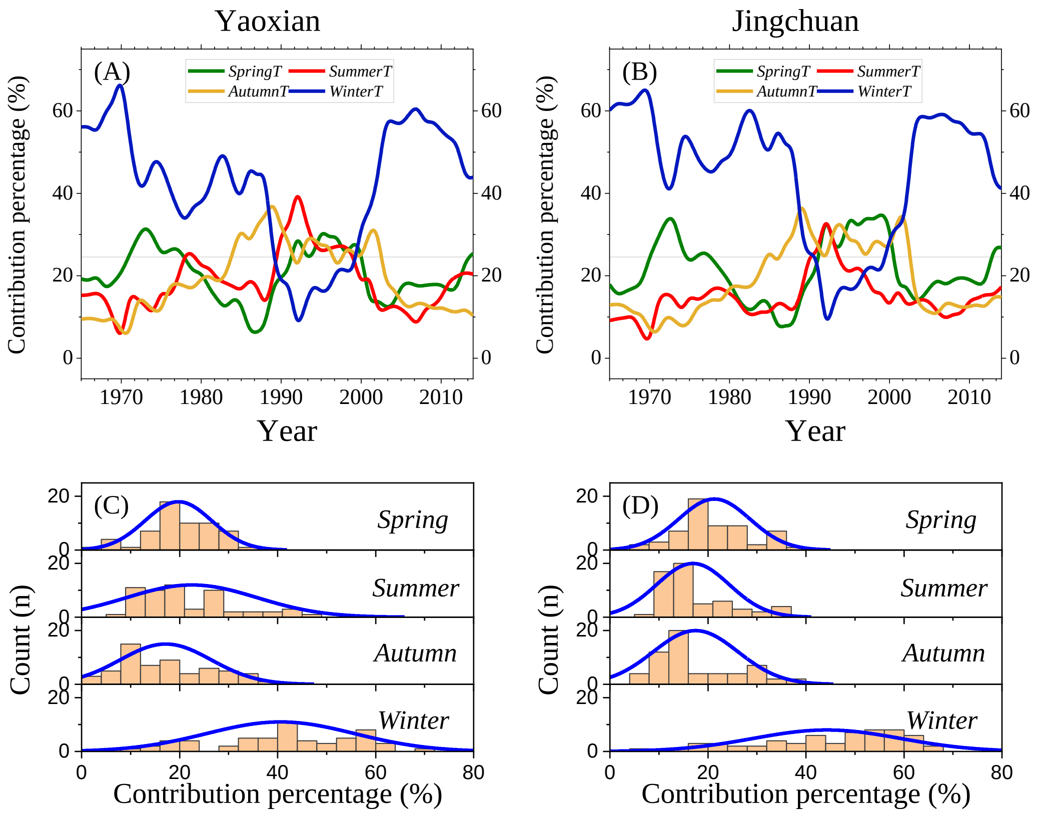

Supplementary Fig. 14. Variation of the relative contribution of mean temperature in different seasons to the MAT over time at Yaoxian (A) and Jingchuan (B) over the past 50 years, and the frequency distribution of explanatory percentages showing the contribution of seasonal temperatures to the reconstructed MAT variation from Yaoxian (C) and Jianchuan (D) sites. The numbers on the horizontal axis are the percentage contribution of each season. The blue line represents the normal distribution curve.

Supplementary Table 1. Descriptive statistics of the environmental variables involved in the modern mollusk datasets.

Variables	Mean	Standard deviation	Minimum	Maximum
MAT (°C)	9.1	3.9	-2.7	17.3
SpringT (°C)	10.1	3.5	-1.6	16.2
SummerT (°C)	21.2	3.3	10.9	27.9
AutumnT (°C)	9.5	3.9	-2.2	18.7
WinterT (°C)	-3.7	5.2	-23	5.9
MAP (mm)	558	181	210	1633
SpringP (mm)	36.3	19.6	9.6	176
SummerP (mm)	101.4	22.5	41.3	177
AutumnP (mm)	44.8	16.8	9.3	91
WinterP (mm)	6.3	7.2	1	59.7
Elevation (m)	1028	652	13	3705
EEE	1595	218	1014	2345
Soil type	11351	232	11000	11925
SUN	2375	318	1479	3103
Vegetation index	0.488	0.15	0.112	0.842

Variable abbreviations: MAT, mean annual temperature; MAP, mean annual precipitation; EEE, Annual evaporation; Soil type is global soil parameters from the Harmonized World Soil Database (http://webarchive.iiasa.ac.at/Research/LUC/External-World-soil-database/HTML/); SUN, sunlight time; Vegetation cover index represented by the NDVI (normalized difference vegetation index).

Supplementary Table 2. Percentage variance of the mollusk species data of the training dataset in northern China explained by each variable in separate canonical correspondence analyses (CCAs), and the percentage of the independent variation explained in a single CCA for all variables together, using the forward selection procedure. Spearman's correlation coefficients (*rS*) between environmental variables and site scores on the first two CCA axes are also shown. The significance (*p*) of the

explained variation (Expl. var.) was tested using a Monte Carlo test with 1999 runs. The order of the variables is based on the amount of explained variation during forward selection.

Variables	Separated CCAs		CCA with forward selection			
	Expl. var. (%)	*p*	Expl. var. (%)	*p*	*rS-Axis 1*	*rS-Axis 2*
MAP (mm)	38.7	0.002	38.7	0.002	0.742	-0.196
SpringT (°C)	22.1	0.002	16.5	0.002	0.352	-0.621
SpringP (mm)	17.8	0.002	8.8	0.002	0.642	-0.122
WinterT (°C)	22.4	0.002	8.3	0.002	0.413	-0.520
SummerT (°C)	22.0	0.002	7.8	0.002	0.358	-0.597
MAT (°C)	18.0	0.002	7.5	0.004	0.207	-0.623
SUN	24.8	0.002	5.8	0.002	-0.564	0.112
Elevation (m)	13.3	0.002	5.7	0.002	-0.446	0.361
AutumnT (°C)	26.1	0.002	5.6	0.018	0.454	-0.585
SummerP (mm)	17.3	0.002	5.3	0.002	0.653	0.003
Vegetation	23.3	0.002	5.2	0.006	0.553	0.053
AutumnP (mm)	13.2	0.002	4.6	0.002	0.529	-0.109
WinterP (mm)	18.7	0.002	4.4	0.002	0.672	-0.230
Soil type	13.2	0.002	4.3	0.004	0.376	0.150
EEE	11.0	0.002	4.0	0.008	-0.294	-0.277

Variable abbreviations: MAT, mean annual temperature; MAP, mean annual precipitation; EEE, Annual evaporation; Soil type is global soil parameters from the Harmonized World Soil Database; SUN, sunlight time; Vegetation cover index represented by the NDVI (normalized difference vegetation index).

Supplementary Table 3. Spearman's correlation coefficients between environmental variables and site scores on the first three principal coordinates analysis (PCoA) axes, demonstrating the main drivers of the observed variation in the modern mollusk data.

Variables	PCoA Axis one	PCoA Axis two	PCoA Axis three

	Spearman's correlation	*P*	Spearman's correlation	*p*	Spearman's correlation	*p*
MAP (mm)	0.691**	<0.001	0.199**	<0.001	-0.030	0.586
WinterT (°C)	0.511**	<0.001	0.208**	<0.001	-0.213**	<0.001
AutumnT (°C)	0.415**	<0.001	0.284**	<0.001	-0.223**	<0.001
Vegetation	0.564**	<0.001	0.174**	0.002	0.056	0.311
MAT (°C)	0.394**	<0.001	0.291**	<0.001	-0.235**	<0.001
SpringT (°C)	0.354**	<0.001	0.271**	<0.001	-0.244**	<0.001
SummerT (°C)	0.283**	<0.001	0.299**	<0.001	-0.204**	<0.001
SpringP (mm)	0.545**	<0.001	0.131*	0.012	0.103*	0.048
SummerP (mm)	0.535**	<0.001	-0.050	0.339	-0.281**	<0.001
AutumnP (mm)	0.499**	<0.001	-0.185**	<0.001	0.095	0.069
WinterP (mm)	0.521**	<0.001	-0.108*	0.040	-0.024	0.652
SUN	-0.516**	<0.001	0.151**	0.004	-0.030	0.561
EEE	-0.353**	<0.001	-0.092	0.078	-0.075	0.152
Elevation	-0.353**	<0.001	-0.092*	0.078	-0.075**	0.152
Soil type	0.265**	<0.001	-0.090**	0.103	-0.045	0.419

**Correlation is significant at the 0.01 level (2-tailed).

Variable abbreviations: MAT, mean annual temperature; MAP, mean annual precipitation; EEE, Annual evaporation; Soil type is global soil parameters from the Harmonized World Soil Database; SUN, sunlight time; Vegetation cover index represented by the NDVI (normalized difference vegetation index).

Supplementary Table 4. Error estimates for reconstruction models of different temperature variables. Model errors are given as the Root Mean Square Errors of Prediction (RMSEP) calculated by bootstrapping (boots). The adjusted coefficients of determination R^2 between boots predicted and observed values, average and maximum bias are also shown.

Model	Variables	*Sample number*	R^2	RMSEP	Mean bias	Max. bias
LW-WAPLS	SpringT (°C)	324	0.6897	1.9231	0.0594	6.2301
	SummerT (°C)	319	0.6670	1.7770	0.0418	4.0039
	AutumnT (°C)	325	0.7121	2.1026	0.1110	4.3509
	WinterT (°C)	326	0.7018	2.6399	0.1488	6.6709
	MAT (°C)	324	0.6798	2.2116	0.1124	4.3665
WAPLS	SpringT (°C)	324	0.6158	1.6761	0.0573	4.5577
	SummerT (°C)	319	0.6265	1.5956	0.0200	3.2203
	AutumnT (°C)	325	0.6735	1.7466	0.0512	3.0186
	WinterT (°C)	326	0.5896	2.6029	0.1495	7.7241
	MAT (°C)	334	0.7305	2.7941	-0.0273	4.3025
LWWA	SpringT (°C)	324	0.6860	1.9175	0.0693	6.0790
	SummerT (°C)	319	0.6660	1.7794	0.0101	3.4803
	AutumnT (°C)	325	0.7116	2.1012	0.1192	4.2274
	WinterT (°C)	326	0.7031	2.6304	0.1721	6.4199
	MAT (°C)	340	0.6912	1.9396	0.0921	4.6436
