# Supplementary material for: The Holocene temperature conundrum answered by mollusk records from East Asia
Source: Nat Commun. 2022 Sep 2;13:5153. doi: 10.1038/s41467-022-32506-7 (PMC9440108; doi:10.1038/s41467-022-32506-7)
Supplement: Supplementary file 2 — Reporting Summary [file 41467_2022_32506_MOESM2_ESM.pdf]

nature portfolio

Corresponding author(s): Yajie Dong; Naiqin Wu

Last updated by author(s): Jun 22, 2022

Reporting Summary

Nature Portfolio wishes to improve the reproducibility of the work that we publish. This form provides structure for consistency and transparency in reporting. For further information on Nature Portfolio policies, see our Editorial Policies and the Editorial Policy Checklist.

Statistics

For all statistical analyses, confirm that the following items are present in the figure legend, table legend, main text, or Methods section.

n/a	Confirmed	
☐	☒	The exact sample size (*n*) for each experimental group/condition, given as a discrete number and unit of measurement
☐	☒	A statement on whether measurements were taken from distinct samples or whether the same sample was measured repeatedly
☐	☒	The statistical test(s) used AND whether they are one- or two-sided<br>*Only common tests should be described solely by name; describe more complex techniques in the Methods section.*
☐	☒	A description of all covariates tested
☒	☐	A description of any assumptions or corrections, such as tests of normality and adjustment for multiple comparisons
☐	☒	A full description of the statistical parameters including central tendency (e.g. means) or other basic estimates (e.g. regression coefficient) AND variation (e.g. standard deviation) or associated estimates of uncertainty (e.g. confidence intervals)
☒	☐	For null hypothesis testing, the test statistic (e.g. *F*, *t*, *r*) with confidence intervals, effect sizes, degrees of freedom and *P* value noted<br>*Give P values as exact values whenever suitable.*
☒	☐	For Bayesian analysis, information on the choice of priors and Markov chain Monte Carlo settings
☒	☐	For hierarchical and complex designs, identification of the appropriate level for tests and full reporting of outcomes
☒	☐	Estimates of effect sizes (e.g. Cohen's *d*, Pearson's *r*), indicating how they were calculated

Our web collection on statistics for biologists contains articles on many of the points above.

Software and code

Policy information about availability of computer code

Data collection	No software was used to collect data.
Data analysis	Program R, version 4.1.3, was used to analyze the data in this study . The code to analyze all the results have been deposited in the Zenodo repository [https://doi.org/10.5281/zenodo.6426798].

For manuscripts utilizing custom algorithms or software that are central to the research but not yet described in published literature, software must be made available to editors and reviewers. We strongly encourage code deposition in a community repository (e.g. GitHub). See the Nature Portfolio guidelines for submitting code & software for further information.

Data

Policy information about availability of data

All manuscripts must include a data availability statement. This statement should provide the following information, where applicable:

- Accession codes, unique identifiers, or web links for publicly available datasets
- A description of any restrictions on data availability
- For clinical datasets or third party data, please ensure that the statement adheres to our policy

The data to support all the analysis in this study have been deposited in the Zenodo repository [https://doi.org/10.5281/zenodo.6426911].

Human research participants

Policy information about studies involving human research participants and Sex and Gender in Research.

Reporting on sex and gender	n/a
Population characteristics	n/a
Recruitment	n/a
Ethics oversight	n/a

Note that full information on the approval of the study protocol must also be provided in the manuscript.

Field-specific reporting

Please select the one below that is the best fit for your research. If you are not sure, read the appropriate sections before making your selection.

☐ Life sciences ☐ Behavioural & social sciences ☒ Ecological, evolutionary & environmental sciences

For a reference copy of the document with all sections, see nature.com/documents/nr-reporting-summary-flat.pdf

Ecological, evolutionary & environmental sciences study design

All studies must disclose on these points even when the disclosure is negative.

Study description	382 surface soil mollusk samples were systematically investigated to establish a transfer function based on the modern mollusk-climate datasets. Then the mollusk-climate calibration model was applied to two fossil mollusk records spanning the last 20,000 years from the Chinese Loess Plateau (CLP), which represents the history of annual and seasonal temperature changes in the East Asian monsoon region.
Research sample	The research samples included 382 topsoil snail assemblages from northern China and 230 fossil snail assemblages from two loess profiles spanning the last 20,000 years.
Sampling strategy	Individual topsoil snails were selected using a combination of a visual search and extraction from a 2–3 cm-thick sample of litter and soil at each site. Fossil snail shells were taken from loess deposits, and all samples were washed and sieved in the field using a 0.5-mm mesh sieve to remove fine soil. The snail shells were all picked and counted.
Data collection	Y. Dong, N. Wu, F. Li, and H. Lu collected the snail data; Y. Dong, F. Li, D. Zhang, and Y. Zhang identified the snail species.
Timing and spatial scale	Surface soil snail samples were mainly collected between 2005 and 2019. Data of fossil snails were collected during 2010 and 2011. The spatial scale of sampling is based on site scale, and the total spatial scale is the regional scale of middle latitude in East Asia.
Data exclusions	No data were excluded from the analyses.
Reproducibility	We identified and counted all of the snail shells. There is no need to repeat counting. The data analysis in this study was conducted with code, and necessary data are included and deposited in the Zenodo repository, so that it is fully reproducible.
Randomization	The sampling sites of surface soil snails were roughly evenly distributed in space and randomly selected to represent the main climatic zones, topography, landscape and vegetation types within the study area. The locations of the fossil snail samples are covered within the range of the topsoil samples, and the sampling profiles are selected in the "Yuan" loess area (flat-topped loess highlands, covered with thick loess deposits), and there are abundant fossil snail shells preserved.
Blinding	Full blinding is not used during surface soil sample collection, but proper blinding is used during data analysis and quantitative reconstruction. Sampling of surface soil snails cannot be fully blinding because we need to know the location of samples, provenience information, climate condition, etc.

Did the study involve field work? ☒ Yes ☐ No

Field work, collection and transport

Field conditions	Field work is not restricted by climatic conditions and can theoretically be done at any time of the year.

Location	The surface soil samples (29.33–49.25°N and 90.22–130.09°E; 13–3705 m above sea level) mainly cover almost the whole Chinese Loess Plateau (CLP), the North China Plain and the surrounding areas. The two loess-palaeosol sections of Yaoxian (34°53′N, 108°58′E, 673 m a.s.l.) and Jingchuan (35°15′N, 107°43′E, 1244 m a.s.l.) are located in the southern and central CLP.
Access & import/export	Our samples were collected with permission, following local and national laws.
Disturbance	Our field sampling will not disturb the natural landscape and environment.

Reporting for specific materials, systems and methods

We require information from authors about some types of materials, experimental systems and methods used in many studies. Here, indicate whether each material, system or method listed is relevant to your study. If you are not sure if a list item applies to your research, read the appropriate section before selecting a response.

Materials & experimental systems

n/a	Involved in the study
☒	☐ Antibodies
☒	☐ Eukaryotic cell lines
☐	☒ Palaeontology and archaeology
☒	☐ Animals and other organisms
☒	☐ Clinical data
☒	☐ Dual use research of concern

Methods

n/a	Involved in the study
☒	☐ ChIP-seq
☒	☐ Flow cytometry
☒	☐ MRI-based neuroimaging

Palaeontology and Archaeology

Specimen provenance	All shell samples were collected independently by our research team. Sampling is permitted following local and national laws, and does not disturb the environment and natural landscape, therefore it does not require any official issuing authority and permission.
Specimen deposition	The shell samples were stored in the Key Laboratory of Cenozoic Geology and Environment, Institute of Geology and Geophysics, Chinese Academy of Sciences, China, and are available to researchers for relevant research purposes upon request.
Dating methods	No new dates are provided.

☒ Tick this box to confirm that the raw and calibrated dates are available in the paper or in Supplementary Information.

Ethics oversight	No ethical approval was required for the non-conflict-of-interest shell samples collected by our own research.

Note that full information on the approval of the study protocol must also be provided in the manuscript.
